# Double Feature Extraction Method of Ship-Radiated Noise Signal Based on Slope Entropy and Permutation Entropy

**DOI:** 10.3390/e24010022

**Published:** 2021-12-23

**Authors:** Yuxing Li, Peiyuan Gao, Bingzhao Tang, Yingmin Yi, Jianjun Zhang

**Affiliations:** 1 School of Automation and Information Engineering, Xi’an University of Technology, Xi’an 710048, China; liyuxing@xaut.edu.cn (Y.L.); 2210320082@stu.xaut.edu.cn (P.G.); 2210321205@stu.xaut.edu.cn (B.T.); yiym@xaut.edu.cn (Y.Y.); 2 School of Electrical Engineering and Automation, Henan Polytechnic University, Jiaozuo 454000, China

**Keywords:** ship-radiated noise signal, permutation entropy, dispersion entropy, fluctuation dispersion entropy, reverse dispersion entropy, slope entropy, feature extraction

## Abstract

In order to accurately identify various types of ships and develop coastal defenses, a single feature extraction method based on slope entropy (SlEn) and a double feature extraction method based on SlEn combined with permutation entropy (SlEn&PE) are proposed. Firstly, SlEn is used for the feature extraction of ship-radiated noise signal (SNS) compared with permutation entropy (PE), dispersion entropy (DE), fluctuation dispersion entropy (FDE), and reverse dispersion entropy (RDE), so that the effectiveness of SlEn is verified, and SlEn has the highest recognition rate calculated by the k-Nearest Neighbor (KNN) algorithm. Secondly, SlEn is combined with PE, DE, FDE, and RDE, respectively, to extract the feature of SNS for a higher recognition rate, and SlEn&PE has the highest recognition rate after the calculation of the KNN algorithm. Lastly, the recognition rates of SlEn and SlEn&PE are compared, and the recognition rates of SlEn&PE are higher than SlEn by 4.22%. Therefore, the double feature extraction method proposed in this paper is more effective in the application of ship type recognition.

## 1. Introduction

Information technology is developing rapidly nowadays, and it is applied in various fields. As an important part of Earth, the investigation, protection, and exploitation of the ocean also needs to be realized with the help of information technology [1,2]. Due to the complexity of marine terrain and the diversity of marine life, and the processive development of underwater noise reduction technology [3,4], the recognition technology of underwater acoustic signal should also be improved. The most important part of the technology is the feature extraction of underwater acoustic signal. It lays a foundation for the further research of underwater acoustic signal positioning, tracking, and detection. As an important kind of underwater acoustic signal, the feature extraction of ship-radiated noise signal (SNS) is key to the development of coastal defenses.

The traditional feature extraction methods of SNS usually take frequency, energy, spectrum and so on as the main feature [5,6,7,8,9]. Frequency-based feature extraction usually needs to be combined with the signal decomposition algorithm, and the intrinsic mode function (IMF) [10] obtained by the signal decomposition algorithm is used as the object of frequency feature extraction, where the signal decomposition algorithms include variational mode decomposition (VMD) [11,12], empirical mode decomposition (EMD) and its improved algorithms [13,14], and the frequency feature includes center frequency and line spectrum frequency. For energy feature extraction of SNS, there are some feature extraction methods based on the improved EMD algorithm and on different energy features, where the algorithms include complete ensemble EMD with adaptive noise (CEEMDAN) and selective noise-assisted EMD (SN-EMD) [15,16,17,18], and the features include energy entropy and energy difference. In the area of spectrum feature extraction, cepstrum, high order spectrum, and continuous spectrum are taken as the main feature. There are also many other kinds of feature, such as chaos feature, correlation dimension, time-frequency feature, etc. [19].

In recent years, some complexity-based feature extraction methods of SNS have been proposed, and tremendous amounts of research show that they are more efficient than traditional feature extraction methods. The complexity features include permutation entropy (PE) [20], dispersion entropy (DE) [21] and its improved algorithms, such as fluctuation dispersion entropy (FDE) [22,23] and reverse dispersion entropy (RDE) [24]. In 2002, PE was proposed for the first time; its advantages are simplicity, exceedingly fast calculation, robustness, etc. [25,26,27]. With the development of PE, it has gradually become more widely used in the field of SNS feature extraction, and the improved algorithms of PE were proposed and applied successively in the following years, such as reverse permutation entropy (RPE) [28], weighted-permutation entropy (W-PE) [29,30], and multi-scale permutation entropy (MPE) [31,32]. In 2016, DE was proposed for the first time; this can quantify the uncertainty of time series, detect the noise bandwidth and simultaneous frequency and amplitude change [33]. In the next three years, some improved algorithms (MDE, FDE, and RDE) of DE were proposed, and their performances for SNS feature extraction have proved better than DE [34,35].

In 2019, David Cuesta-Frau [36] proposed a new entropy estimator termed Slope entropy (SlEn), which is based on the relative frequency of simple symbol patterns. She used SlEn to extract electroencephalographic (EEG) signals compared with PE and SE, and the results show that SlEn has the best classification effect. In recent years, SlEn has been further developed and applied. In September 2020, David Cuesta-Frau et al. [37] proposed a method based on SlEn for distinguishing differences in body temperature records deriving from various classes of disease backgrounds, and their conclusions indicate that SlEn has a high ability to discriminate the temperature record sequences of different patients with dengue, malaria, a malignant tumor, and leptospirosis. In November 2020, David Cuesta-Frau et al. [38] designed a study based on SlEn to compare dynamic recordings from internal emotional outburst symptoms of long follow-up patients with bipolar disorder (BD), and the results proved that SlEn is practicable for easily distinguishing between depression and mania episodes. These papers prove that SlEn is an entropy estimator with a good classification effect. However, SlEn has not been applied to underwater acoustic signal processing.

In this paper, SlEn is introduced into the feature extraction of SNS for the first time. We propose a single feature extraction method based on SlEn and a double feature extraction method based on SlEn&PE. The rest of the paper is as follows: Section 2 introduces the basic principle of SlEn and gives an example of the algorithm. In Section 3, two feature extraction methods are proposed, and the detailed steps are introduced. Section 4 and Section 5 carry out the experiments of single feature extraction and double feature extraction, and the classification based on k-Nearest Neighbor (KNN). Section 6 offers a summary of this paper and describes its main innovations and conclusions.

## 2. Slope Entropy

**Figure 1:**
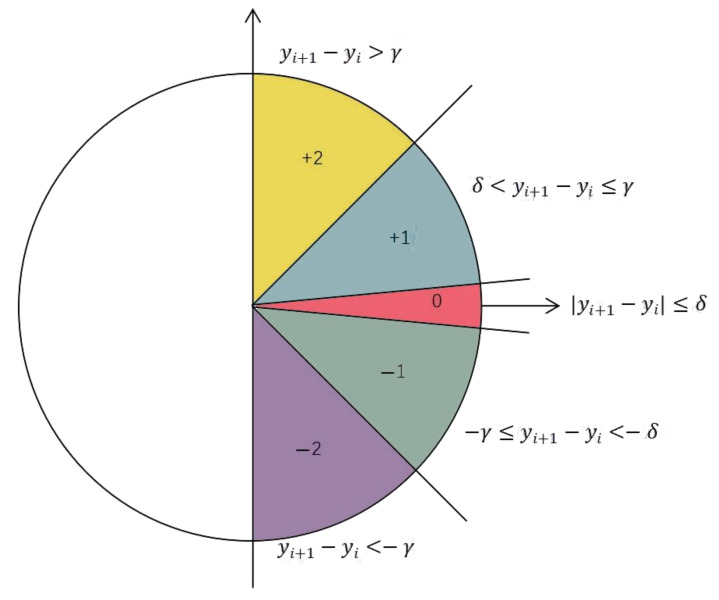
Symbol assignment of SlEn.

Slope entropy (SlEn) is an algorithm proposed in 2019 which can represent the complexity of time series. It is based both on symbolic patterns and amplitude information. Each symbol is set up by the difference between continuous samples of the input time series. The SlEn algorithm has only five symbol patterns, which makes it easy to implement. The calculation process of SlEn is as follows [35]:

(1) For given time series $Y=\left\{y_{i},\;i=1,\;2,\;\ldots \;,\;N\right\}$, the sub-sequences of Y are extracted according to the embedding dimension m, $Y_{1}=\left\{y_{1},\;y_{2},\;\ldots \;,\;y_{m}\right\}$, $Y_{2}=\left\{y_{2},\;y_{3},\;\ldots \;,\;y_{m+1}\right\}$, ... , $Y_{k}=\left\{y_{k},\;y_{k+1},\;\ldots \;,\;y_{N}\right\}$, where k=N−m+1.
(2) Two threshold parameters (γ  and δ) are used to divide different symbol patterns (+2, +1, 0, −1, −2). Figure 1 is symbol assignment of SlEn.

The specific symbol patterns of SlEn distribution are very explicit. SlEn considers the horizontal increment between consecutive samples to be always 1, and the vertical increments are divided by γ  and δ. If γ=1, the slope of the boundaries is 45° and −45°. And the region of symbol “0” is determined by threshold parameter δ.

The specific distribution principle is as follows: if $y_{i+1}-y_{i}>\gamma$, the symbol pattern is +2; if $\delta <y_{i+1}-y_{i}\leq \gamma$, the symbol pattern is +1; if $\left|y_{i+1}-y_{i}\right|\leq \delta$, the symbol pattern is 0; if $-\gamma \leq y_{i+1}-y_{i}<-\delta$, the symbol pattern is −1; if $y_{i+1}-y_{i}<-\gamma$, the symbol pattern is −2, where γ>δ>0.

(3) Pattern sequences $S_{1},\;S_{2},\;\ldots \;,\;S_{k}$, which correspond to $Y_{1},\;Y_{2},\;\ldots \;,\;Y_{k}$, are obtained after symbol assignment, $S_{1}=\left\{s_{1},\;s_{2},\;\ldots \;,\;s_{m-1}\right\}$, $S_{2}=\left\{s_{2},\;s_{3},\;\ldots \;,\;s_{m}\right\}$, ..., $S_{k}=\left\{s_{k},\;s_{k+1},\;\ldots \;,\;s_{N-1}\right\}$, where k=N−m+1, $s_{1},\;s_{2},\;\ldots \;,\;s_{N-1}$ are the symbol patterns obtained by $y_{2}-y_{1}$, $y_{3}-y_{2}$, ..., $y_{N}-y_{N-1}$ through step (2).
(4) Pattern sequence has $n=5^{m-1}$ different types. The number of each type is $k_{1},\;k_{2},\;\ldots \;,\;k_{n}$. The relative frequency of the sequences are their probabilities: $P_{1}=\frac{k_{1}}{k}$, $P_{2}=\frac{k_{2}}{k}$, ..., $P_{n}=\frac{k_{n}}{k}$. Based on the classical Shannon entropy, the definition formula of SlEn is obtained as follows:

(1) $$H_{s}\left(m\right)=-\overset{n}{\underset{j=1}{\sum }}P_{j}\ln P_{j}$$

## 3. Proposed Feature Extraction Methods

In this experiment, a single feature extraction method and a double feature extraction method are proposed for SNS.

**Figure 2:**
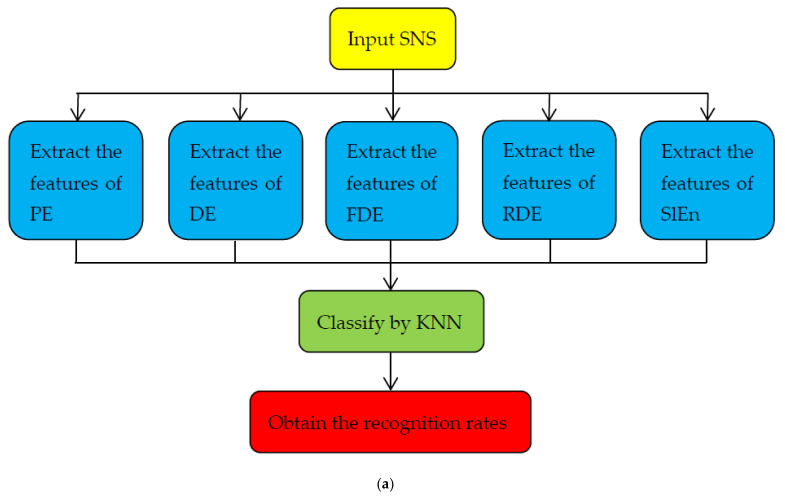

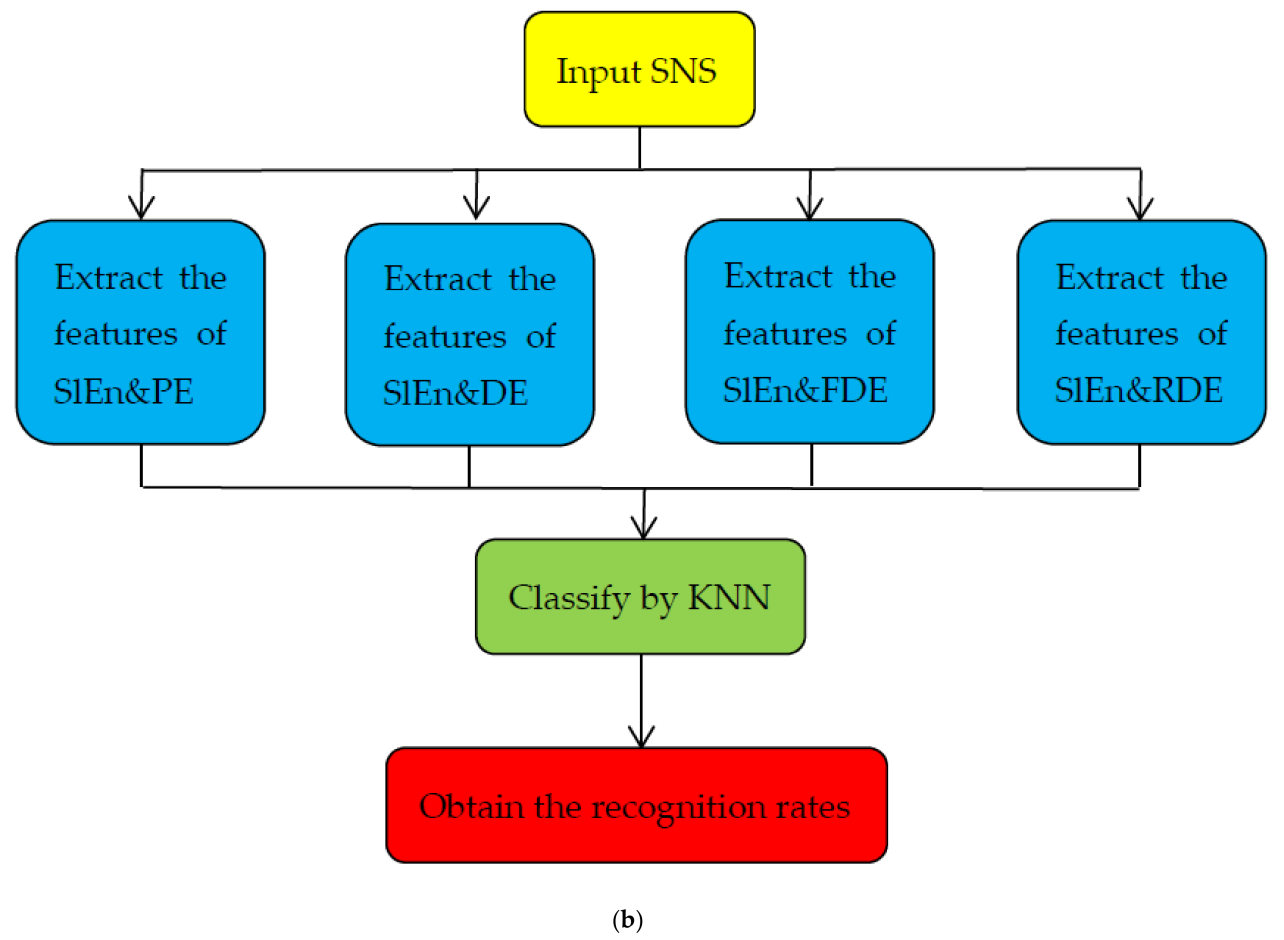
The flow chart of feature extraction methods. (**a**) Single feature, (**b**) double feature.

As shown in Figure 2a, the specific steps of single feature extraction method are as follows:

(1) The four types of normalized SNS are inputted.
(2) For each type of normalized SNS, 500 samples are selected and five features are extracted, including PE, DE, FDE, RDE, and SlEn.
(3) K-Nearest Neighbor (KNN) is used to classify the four types of ship signals, and we set the number of nearest samples as k=1. For each type, select 50 sample signals as training samples and 450 sample signals as test samples.
(4) The recognition rate of SNS can now be obtained. By comparing the recognition rates formed by SlEn and other four different kinds of entropy, we can know the validity of SlEn in the classification of single feature.

Figure 2b shows a flow chart for the double feature extraction method. The only difference between this and the single feature extraction method is step (2). The double features are slope entropy combined with permutation entropy (SlEn&PE), slope entropy combined with dispersion entropy (SlEn&DE), slope entropy combined with fluctuating dispersion entropy (SlEn&FDE), and slope entropy combined with reverse dispersion entropy (SlEn&RDE). Moreover, by comparing the recognition rates formed by SlEn&PE and three other kinds of combined entropy, we can know the validity of SlEn&PE in the classification of double feature.

## 4. Single Feature Extraction of SNS

### 4.1. Four Types of SNS

**Figure 3:**
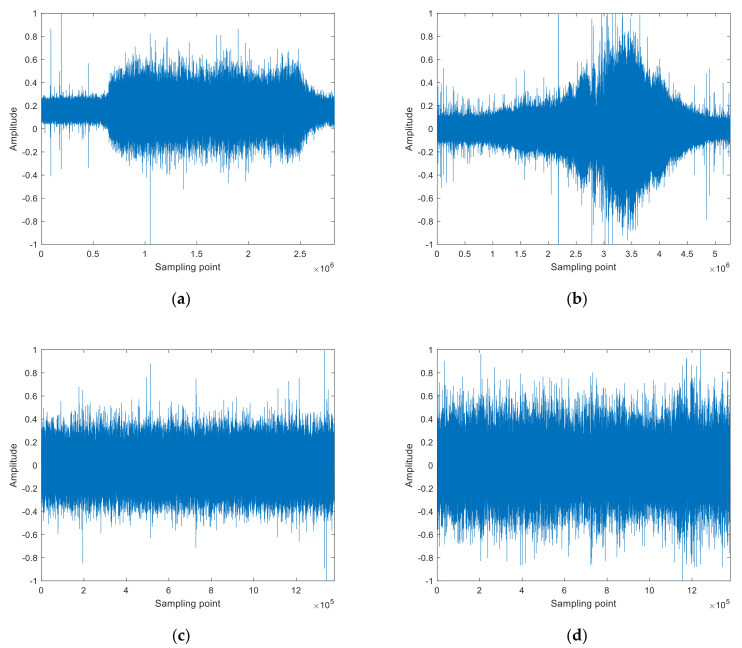
The normalized four types of SNS. (**a**) Ship–①, (**b**) ship–②, (**c**) ship–③, (**d**) ship–④.

Single feature extraction is implemented for four types of measured SNS, termed ship–①, ship–②, ship–③, and ship–④. Ship–① and ship–② derive from the same website [39] and represent an ocean liner and a motorboat, respectively. Ship–③ and ship–④ derive from another website [40] and represent an Alaska state ferry and a cruise ship, respectively. The length of sampling point for ship–① is 2,828,835, the sampling length for ship–② is 5,269,916, and the sampling length for ship–③ and ship–④ is 1,380,000. The sampling frequency of ship–① and ship–② is 52,734 Hz, and the sampling frequency of ship–③ and ship–④ is 44,100 Hz. The normalized four types of SNS are shown in Figure 3.

### 4.2. Single Feature Extraction

**Figure 4:**
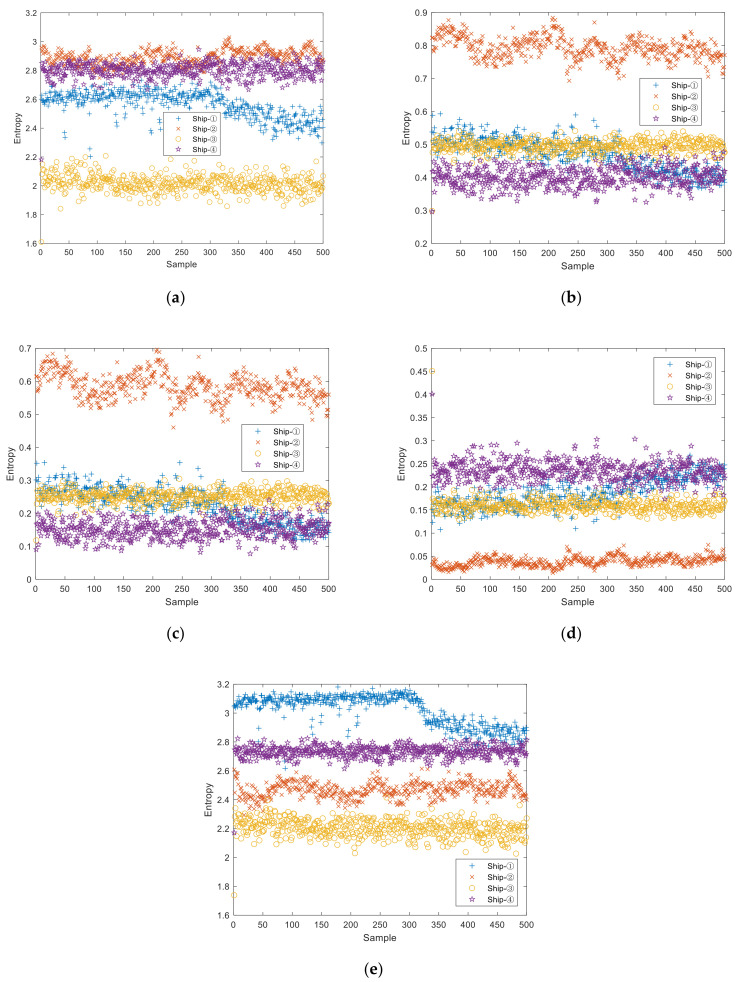
Single feature distribution of four types of SNS. (**a**) PE, (**b**) DE, (**c**) FDE, (**d**) RDE, (**e**) SlEn.

For each type of SNS, 500 samples are selected in the single feature extraction experiment, with each sample containing 2000 sampling points. For the sake of comparison, we set the embedding dimension as m=4, the delay time as τ=1, the number of categories of DE, FDE, and RDE as *c* = 3, and the two threshold parameters of SlEn as γ=1 and δ=0.001, with the mapping formats of DE and FDE as the normal cumulative distribution function (NCDF). Single feature distribution of four types of SNS is shown in Figure 4.

It can be seen from Figure 4 that, for PE distribution, the entropies of ship–①, ship–②, and ship–④ are close to each other; that, for DE, FDE, and RDE distribution, the entropies of ship–①, ship–③, and ship–④ are similar to each other; that, for SlEn distribution, only a few entropies of ship–① approach equality with a few entropies of ship–④. It can be concluded that SlEn has better inter-class separability for the four types of SNS.

In order to prove the validity of SlEn, the mean, minimum mean difference (MMD), and coefficient variations (CV) of different features are calculated. MMD is the absolute value of the smallest mean difference. CV is the ratio of the standard deviation to the mean, and when the CV is smaller, the smaller the dispersion degree and the more stable it is. Table 1 shows the mean, MMD and CV of different features.

As shown in Table 1, for these five kinds of entropy, the mean value of each type of SNS has different degrees of difference. DE has the smallest MMD of 0.025, and the MMD of SlEn is the biggest with 0.2652. For ship–①, the CV of PE and the CV of SlEn are very close and are far smaller than the CV of DE, FDE, and RDE; for ship–②, PE has the smallest, with a CV of 0.0175, while the CV of SlEn is 0.0216, making them far smaller than the CV of DE, FDE and RDE; for ship–③ and ship–④, SlEn has the two smallest CV of 0.0289 and 0.0165, respectively. It shows that the MMD of SlEn is the biggest, while SlEn has the smaller CV. Therefore, SlEn has the best separability and stability.

### 4.3. Single Feature Classification

**Figure 5:**
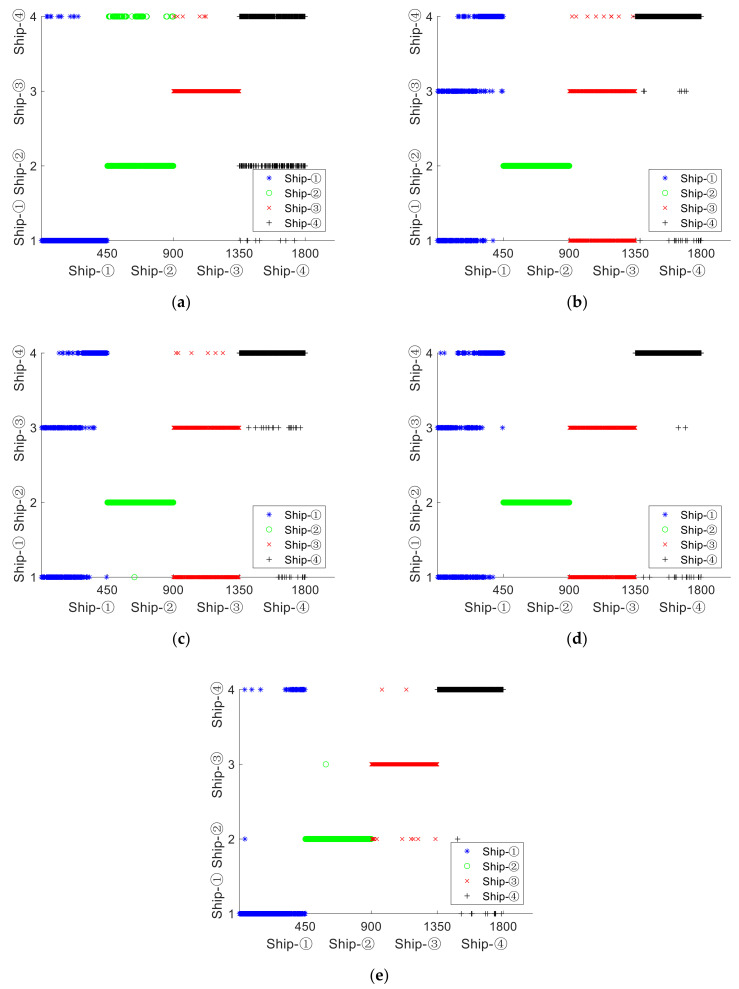
Single feature classification and recognition distribution. (**a**) PE, (**b**) DE, (**c**) FDE, (**d**) RDE, (**e**) SlEn.

In order to prove that the single feature extraction of SNS based on SlEn is better, KNN classification is introduced in this experiment. Select 500 samples for the four types of SNS, respectively, with the first 50 samples taken as training samples, and the other 450 samples classified as test samples. The single feature classification and recognition distribution is shown in Figure 5.

Figure 5 shows that PE has different numbers of wrongly classified samples for the four types of SNS, of which ship–④ has the largest number of wrongly classified samples; DE, FDE, and RDE show the samples of ship–② to be basically correct, but there are lots of wrongly classified samples for ship–① and ship–③; SlEn has a few wrongly classified samples for the four types of SNS, with only one wrongly classified sample for ship–②; for the five kinds of extraction methods, the classification effect of SlEn&PE is the best on average. The recognition rates of single feature are shown in Table 2.

As shown in Table 2, for ship–①, PE has the highest recognition rate of 97.33%, the recognition rate of SlEn is close to 90%, and the recognition rates of DE, FDE, and RDE for ship–① are less then 40%; for Ship–②, the recognition rates are all more than 99% except PE; for Ship–③, the recognition rates of PE and SlEn are more than 97%, and DE, FDE, and RDE have recognition rates of less than 60%; for ship–④, SlEn has the highest recognition rate of 97.56%, and PE has the lowest recognition rate of 71.56%; for the four types of SNS, SlEn has the highest average recognition rate of 95.72%. The average recognition rates of the other four features are less than 90%.

It is shown that the classification of SlEn for the four types of SNS samples is the most stable and has the highest average recognition rate. To further improve the recognition rate, we combine PE with SlEn and propose a double feature extraction method.

## 5. Double Feature Extraction of SNS

### 5.1. Double Feature Extraction

**Figure 6:**
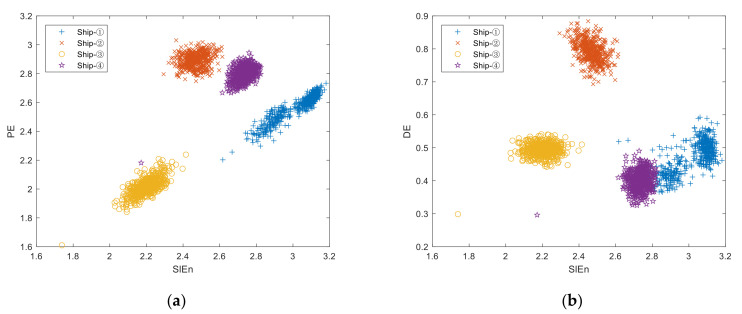

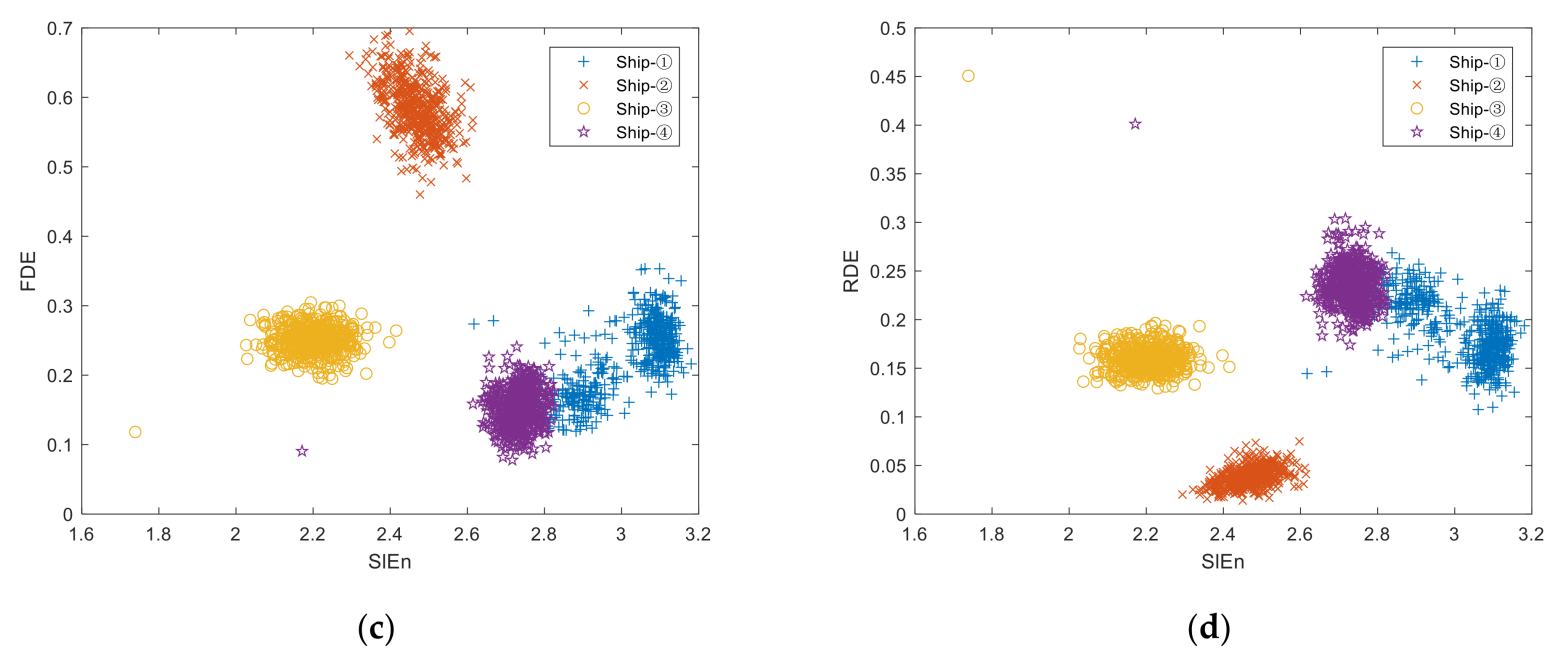
Double feature distribution of four types of SNS. (**a**) SlEn&PE, (**b**) SlEn&DE, (**c**) SlEn&FDE, (**d**) SlEn&RDE.

SlEn has the best recognition rate in single feature extraction, so we use SlEn to combine with the other four kinds of entropy in the double extraction experiment. All parameters used in the experiment are the same as those listed in Section 4.2. The double feature distribution of four types of SNS is shown in Figure 6, where the abscissa represents SlEn, and the ordinate represents the other four kinds of entropy.

It can be seen from Figure 6 that, for SlEn&PE distribution, the points of the entropies of the four kinds of ships are far away from each other; that, for SlEn&DE, SlEn&FDE and SlEn&RDE distribution, some entropies of ship–① are nearly equal to some entropies of ship–④. This indicates that SlEn&PE has better inter-class separability for the four types of SNS.

### 5.2. Double Feature Classification

**Figure 7:**
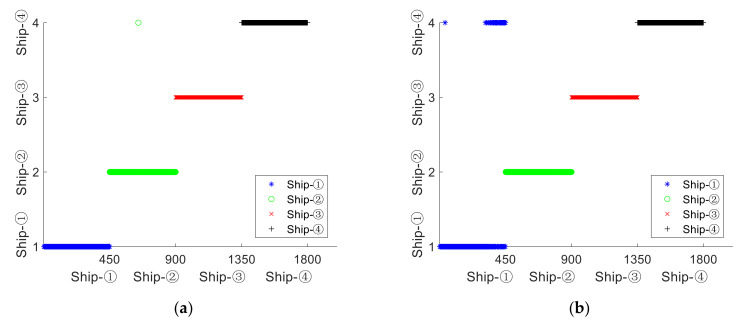

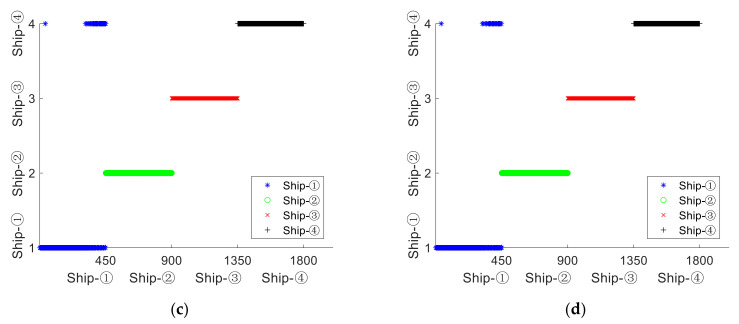
Double feature classification and recognition distribution. (**a**) SlEn&PE, (**b**) SlEn&DE, (**c**) SlEn&FDE, (**d**) SlEn&RDE.

In order to prove that the double feature extraction of SNS based on SlEn&PE is better, KNN classification is also introduced in this experiment. The same number of training samples and test samples as Section 4.3 are used. Figure 7 shows the double feature classification and recognition distribution.

As illustrated in Figure 7, SlEn&PE has only one wrongly classified sample for ship–②; for the other three types of SNS, the classification of the samples is completely correct; SlEn&DE, SlEn&FDE, and SlEn&RDE classify the samples of ship–②, ship–③, and ship–④ completely correctly, while there are many wrongly classified samples when they classify the samples of Ship–①; for the four kinds of extraction methods, SlEn&PE has the best classification effect. The recognition rates of double feature is shown in Table 3.

As shown in Table 3, for ship–①, SlEn&PE has the highest recognition rate of 100%, while the recognition rates of SlEn&DE, SlEn&FDE, and SlEn&RDE are less than 90%; for ship–②, ship–③, and ship–④, the recognition rates are all 100%, except that SlEn&PE has the recognition rate of 99.78% for ship–②; for the four types of SNS, SlEn&PE has the highest average recognition rate of 99.94%. The average recognition rates of the other three features are less than 98%.

It is shown that the classification of SlEn&PE for the four types of SNS samples has the highest average recognition rate, having only one wrongly classified sample in the population, which is 4.22% higher than the proposed single feature extraction method.

### 5.3. Comparison of Different Methods

For further proof of the effectiveness of the method proposed in this paper, some methods from previously published literature are cited by way of comparison. In one, 50 samples are selected from each kind of ship signal to be used for training the classifier while the remaining ones are left to be used for testing the performance. These testing data are fed into the KNN classifier to classify the types of different ships. The classification data and the computing time of different methods are listed in Table 4.

As illustrated in Table 4, the double feature extraction method proposed in this paper has the shortest computing time because it doesn’t use any signal decomposition algorithms. In addition, compared with the other three feature extraction methods, the proposed double feature extraction method has the highest recognition rate, which further proves the effectiveness of the proposed method.

## 6. Conclusions

SlEn is applied to the field of underwater acoustic signal processing, and two feature extraction methods are proposed. The feasibility of the proposed methods is verified by the feature extraction and classification of four kinds of measured SNS. The main innovations and conclusions of this study are as follows:

(1) SlEn is introduced into the feature extraction of SNS for the first time, and a single feature extraction method based on SlEn and a double feature extraction method based on SlEn&PE are proposed.
(2) Compared with the single feature extraction method of SNS based on PE, DE, FDE, and RDE, the proposed single feature extraction method based on SlEn has smaller CV, which proves that SlEn is more stable. Moreover, it has the highest average recognition rate of 95.72%, which is at least 8% higher than the other four single feature extraction methods.
(3) The average recognition rate of the proposed double feature extraction method is 4.22% higher than the proposed single feature extraction method. Compared with the other three double feature extraction methods, the proposed double feature extraction method has the highest average recognition rate of 99.94%.

## Figures and Tables

**Table 1:** The mean, MMD, and CV of different features.

Entropy	Type	Ship–①	Ship–②	Ship–③	Ship–④
PE	Mean	2.563	2.8928	2.0172	2.8007
	MMD	0.0921			
	CV	0.036	0.0175	0.0309	0.0196
DE	Mean	0.47	0.793	0.495	0.4009
	MMD	0.025			
	CV	0.1037	0.0423	0.0402	0.0712
FDE	Mean	0.2251	0.5848	0.2512	0.1522
	MMD	0.0261			
	CV	0.2265	0.0683	0.0791	0.1818
RDE	Mean	0.1874	0.0386	0.1599	0.2358
	MMD	0.0275			
	CV	0.1665	0.2665	0.1115	0.0914
SlEn	Mean	3.0152	2.4674	2.2022	2.7328
	MMD	0.2652			
	CV	0.0367	0.0216	0.0289	0.0165

**Table 2:** Recognition rates of single feature.

Entropy	Ship–① (%)	Ship–② (%)	Ship–③ (%)	Ship–④ (%)	Average (%)
PE	97.33	82	98.67	71.56	87.39
DE	35.11	100	52	94.89	70.5
FDE	37.33	99.78	53.78	93.11	71
RDE	34.89	100	56.44	96.22	71.89
SlEn	88.44	99.78	97.11	97.56	95.72

**Table 3:** Recognition rates of double feature.

Entropy	Ship–① (%)	Ship–② (%)	Ship–③ (%)	Ship–④ (%)	Average (%)
SlEn&PE	100	99.78	100	100	99.94
SlEn&DE	85.78	100	100	100	96.44
SlEn&FDE	85.11	100	100	100	96.28
SlEn&RDE	89.78	100	100	100	97.44

**Table 4:** Classification data and the computing time of different methods.

Method	Recognition Rate (%)				Average (%)	Computing Time (s)
	Ship–①	Ship–②	Ship–③	Ship–④		
VMD-PE	94	100	99.33	100	98.33	5927.7852
CEEMDAN-ED-EE	96.89	88.89	77.78	77.78	85.33	45,202.5006
CEEMDAN-W-PE	97.11	85.33	77.56	77.78	84.44	45,494.1924
SlEn&PE	100	99.78	100	100	99.94	48.6153

## Data Availability

The data used to support the findings of this study are available from the corresponding author upon request.
